# Influence of High Temperature on the Fracture Properties of Polyolefin Fibre Reinforced Concrete

**DOI:** 10.3390/ma14030601

**Published:** 2021-01-28

**Authors:** Marcos García Alberti, Jaime Carlos Gálvez, Alejandro Enfedaque, Ramiro Castellanos

**Affiliations:** Departamento de Ingeniería Civil: Construcción, E.T.S de Ingenieros de Caminos, Canales y Puertos, Universidad Politécnica de Madrid, C/Profesor Aranguren, s/n, 28040 Madrid, Spain; jaime.galvez@upm.es (J.C.G.); alejandro.enfedaque@upm.es (A.E.); ramiro.castellanos@upm.es (R.C.)

**Keywords:** fracture behaviour, fibre reinforced concrete, high temperature, melting point, flexural tensile strength, polyolefin fibres

## Abstract

Concrete has become the most common construction material, showing, among other advantages, good behaviour when subjected to high temperatures. Nevertheless, concrete is usually reinforced with elements of other materials such as steel in the form of rebars or fibres. Thus, the behaviour under high temperatures of these other materials can be critical for structural elements. In addition, concrete spalling occurs when concrete is subjected to high temperature due to internal pressures. Micro polypropylene fibres (PP) have shown to be effective for reducing such spalling, although this type of fibres barely improves any of the mechanical properties of the element. Hence, a combination of PP with steel rebars or fibres can be effective for the structural design of elements exposed to high temperatures. New polyolefin fibres (PF) have become an alternative to steel fibres. PF meet the requirements of the standards to consider the contributions of the fibres in the structural design. However, there is a lack of evidence about the behaviour of PF and elements made of polyolefin fibre reinforced concrete (PFRC) subjected to high temperatures. Given that these polymer fibres would be melt above 250 °C, the behaviour in the intermediate temperatures was assessed in this study. Uni-axial tests on individual fibres and three-point bending tests of PFRC specimens were performed. The results have shown that the residual load-bearing capacity of the material is gradually lost up to 200 °C, though the PFRC showed structural performance up to 185 °C.

## 1. Introduction

One of the most relevant advantages of concrete as a construction and building material, compared with other structural materials such as steel or wood, is its behaviour when subjected to fire and high temperatures. In the 1970s, an extensive experimental campaign initiated to analyse and investigate the behaviour of concrete subjected to high temperatures, due to the growth of the nuclear industry that uses this material as confinement in reactors and for cooling towers [1]. However, concrete is weak in tension and needs to be combined for structural purposes with other materials such as steel. Thus, the behaviour of concrete structures subjected to high temperatures is not only related to the behaviour of concrete itself, but also to the behaviour of the reinforcing materials.

Given that fire remains as one of the most relevant potential risks for structures, research dealing with the response of reinforced concrete and fibre reinforced concrete under this circumstance has been conducted, showing that some micro polypropylene fibres (PP) can be used to control the risk of explosive spalling [2]. Certain types of fibres have shown to enhance the response of the concrete element exposed to fire or high temperatures [3]. The damage caused by high temperatures can be limited due to the increase of pore connectivity of the PP fibres after melting. The pore structure of concrete directly influences the phenomenon of spalling occurring in a controlled or even in a forceful way (explosive spalling), if the pore structure is more closed, as in the case of HSC (high strength concrete). The effect of the internal vapor pressure of the concrete is fundamentally due to the dehydration generated, and the thermal stress due to the thermal gradient that it supports while it is subjected to high temperatures [4]. Therefore, the use of fibres, especially mixing with PP fibres, has shown to improve significantly the properties of the concrete because the high vapor pressure due to the inner moisture in concrete is released by the micro channels left by the PP fibres when are melted [5]. The mixture between steel (SF) and PP fibres helps optimising the residual properties that the concrete exposed to high temperatures could reach [2,6] and takes advantage of the contribution to fire resistance of PP microfibers and the structural reinforcement provided by SF. Thus, the combination of both types of fibres optimises the behaviour under high temperatures [7]. PP fibres can hardly bear any additional loadings (they are not considered as structural fibres), though they melt with high temperatures, creating a capillary network that avoids high pressures inside the element subjected to high temperature, reducing the spalling.

Several types of synthetic fibres such as PP, polyvinyl alcohol (PVA), nylon (Ny), polyethylene (PE) have shown several advantages and have been used mainly in combination with SF and specially for high strength concrete [8]. Steel fibres are the most common structural fibres. Nevertheless, research has shown that concrete reinforced only with SF, regardless of the content, exhibits explosive spalling. Recent advances have shown that polyolefin macro-fibres (PF) can also meet residual tensile strengths that can be considered in structural design to substitute steel rebars [9,10,11]. Such fibres have shown to be an attractive alternative to steel fibres in certain applications [12,13]. However, there is a lack of studies dealing with this type of structural polymer fibres when exposed to high temperature. In such a sense, PF are expected to melt at medium temperatures such as 200 °C. Thus, some additional beneficial effects could appear in terms of spalling if the melting of the fibres leaves channels that help reducing the internal vapor pressure. However, PF are mainly used for structural reinforcement of concrete elements [12,14]. Therefore, the most important characterisation is to assess the residual load bearing capacity of polyolefin fibre reinforced concrete (PFRC) elements exposed to high temperature.

The significance of this research relies on the assessment of the behaviour of PFRC exposed to high temperature. To the authors’ best knowledge, this is the first time that polyolefin fibres have been studied under such conditions. Moreover, the fracture properties and the residual strengths were obtained by three-point bending tests on notched specimens previously exposed to the referred a range of temperature from 20 °C to 200 °C. The results have shown that the residual load-bearing capacity of the material is gradually lost up to 200 °C, though the PFRC showed structural performance up to 185 °C. In addition, in this research, the variation of the mechanical properties of isolated PF exposed to a range of temperature from 20 °C to 160 °C was studied.

## 2. Experimental Program and Results

The experimental campaign encompassed two main research aims: characterisation of the properties of isolated fibres subjected to high temperatures and characterisation of the residual properties of fibre reinforced concrete specimens after being exposed to high temperatures. The specimens were subjected to heat in a Memmet UFB-500 stove (Schwabach, Germany) capable of reaching temperatures above 200 °C with an accuracy of ±0.5 °C

### 2.1. Fibre Characterisation

Isolated polyolefin-based macro fibres were introduced in an oven at various temperatures in order to assess the influence of temperature on the mechanical properties. In order to evaluate such mechanical properties, uniaxial tensile tests were performed supplying values of load and displacements. Digital image techniques (DIC) were implemented to obtain the longitudinal deformation. With such results and with a previous meticulous measurement of the fibre cross section and the initial length, it was possible to compute the ultimate residual strength for each temperature. With the stress–strain curve, the residual modulus of elasticity was calculated as the slope of the fitting curve in the quasi-straight stretch. The fibres tested were Sika-fiber T-60 (Baar, Switzerland) [15] and their main physical and mechanical properties are presented in Table 1.

The fibres were introduced in the oven when it had reached the target temperature for an hour. Then, the fibres were kept in the oven for 24 h. This timing was chosen following the rationale found in references [2,7]. Before testing, the fibres were released from the oven and the loss of mass was measured. The tests were performed at laboratory temperature.

**Figure 1:**
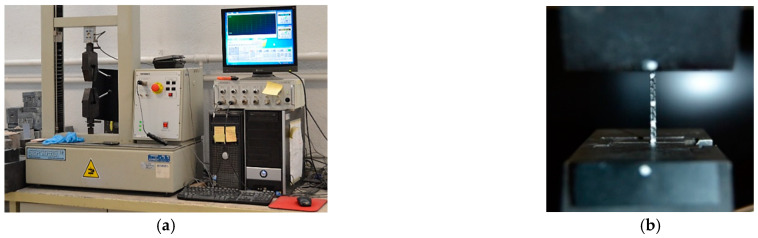
(**a**) Testing machine for fibres characterisation; (**b**) fibre placed for the tensile test.

The uniaxial tensile tests of the fibres were performed according to UNE-EN 6892-1: 2017 [16] assumed valid for polyolefin fibres. The testing equipment used has two jaws for fastening the fibres with a maximum load capacity of 10 kN. An actuator displacement rate of 0.169 mm/s was used. Video extensometry system with a high-definition camera was used. Four white points were painted on the fibres tested, obtaining images at one frame per second rate during the course of the test, determining the elongation of the fibre by synchronising the video with the results of the machine (load and displacement values). This setup can be seen in Figure 1. The two points painted close to the jaws, see Figure 1b, were used to control by DIC techniques that there were not any relative displacements between the fibre and the jaws.

The video recorded one image per second by use of a high-definition IDS UI-1480SE camera (Obersulm, Baden-Württemberg, Germany). This device has a five-megapixel sensor with a resolution of 2560 × 1920 pixels. The application of a two-dimensional image analysis can be obtained by synchronising mechanical testing and video recording.

**Figure 2:**
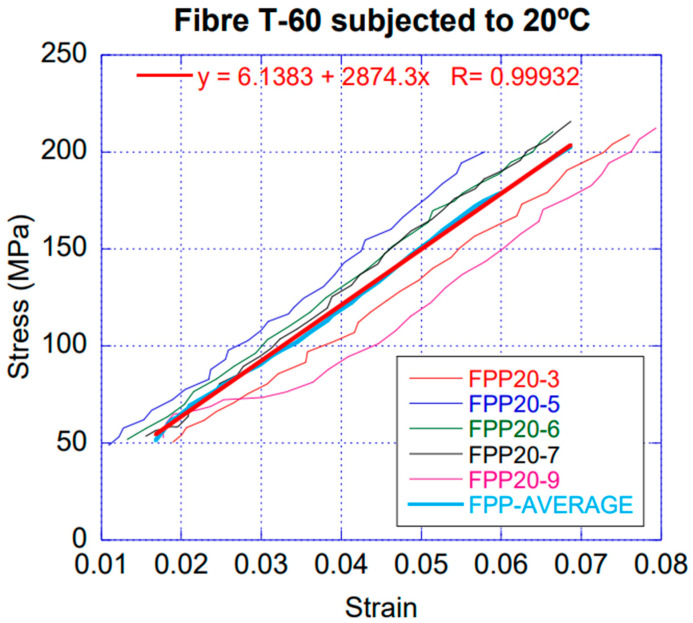
Stress–strain curves of the fibres at 20 °C: individual curve for each specimen (FPP20-#), average curve and linear fitting of the average curve (shown in the legend).

**Figure 3:**
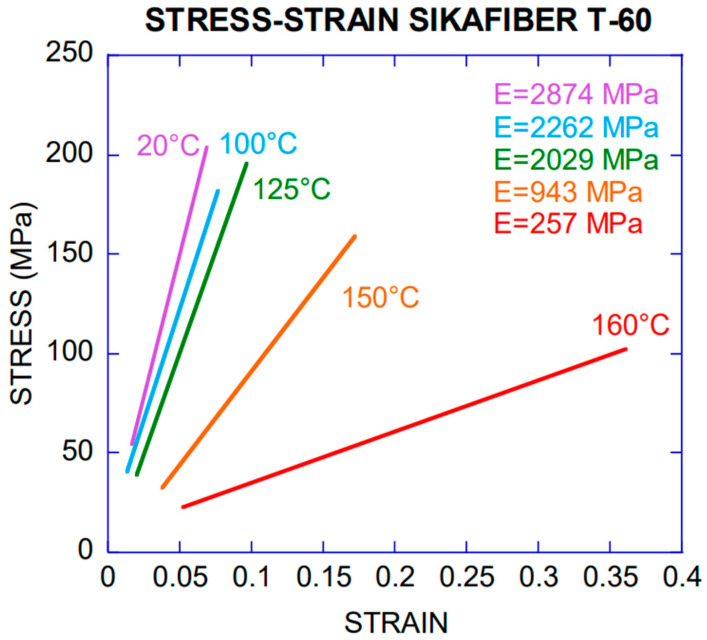
Average stress–strain curves obtained with a linear fitting of five specimens tested after exposed to various temperatures.

The temperatures studied were 100 °C, 125 °C, 150 °C and 160 °C. This set of temperatures permitted assessing the progressive modification of their properties. Higher temperatures would not allow performing the test because of the melting of the fibres. For each one, five specimens were tested, and stress–strain curves were recorded. Given that this type of test in polyolefins may show high degrees of scattering of the experimental results, such number of valid tests was chosen for each temperature. A representative average curve was interpolated for each temperature. Table 2 shows these values and Figure 2 shows an example with the five curves and the average. For the sake of clarity, the comparison showed by Figure 3 only includes the average curves. The figure was performed considering values between 20% and 80% of the maximum fracture or sliding load. Such an interval was considered representative for obtaining the modulus of elasticity given that occasionally the fibre initially can slide and settle gradually in the jaws, shown by a non-linear stress–strain curve at the beginning of the test. The maximum stress for each temperature was obtained as the average from five tests and with the cross section of each fibre measured after being exposed to the correspondent temperature.

Figure 3 shows that the loss of mechanical properties increases with temperature. This reduction was more evident from 150 °C to 160 °C. After being exposed to those temperatures, the modulus of elasticity showed residual values of 33% and 9%, respectively, of that at room temperature as can also be seen in Table 2. It should also be worth noting that the fibres shortened with temperature and, therefore, increased the cross-section and the deformation capacity was considerably higher. Both the cross-section and the length (see Figure 8) were measured when the fibres were again at room temperature after heating.

### 2.2. Characterisation of Fibre Reinforced Concrete

The experimental campaign was performed with concrete elements manufactured with the concrete mix design of the reference [17]. Thus, siliceous crushed aggregates, with a maximum aggregate size of 12.7 mm, were used. Portland cement type EN-197-1 CEM I 52.5 R-SR, polycarboxylic superplasticiser Sika Viscocrete 5720, and limestone powder with a content of 98% calcium carbonate [9,17] were used. Fibre dosages of 3 and 10 kg/m^3^, with a water/cement ratio of 0.5, were employed. The mix proportioning can be seen in Table 3 and it was the same used in references [9,17], so that the values obtained at room temperature could be compared. In addition, plain concrete specimens were also tested in order to compare the results with fibre reinforced concrete.

The characterisation of the concrete elements encompassed three reference temperatures. Given that fibres were supposed to melt for higher temperatures, the highest temperature of exposure was set at 200 °C. Moreover, as the isolated fibres maintained a high degree of integrity for 150 °C, such temperature was also used for the three types of concrete (plain and with 3 and 10 kg/m^3^ of polyolefin fibres). The third temperature was the room temperature in order to compare the residual properties with the original ones. After exposed to those temperatures, the properties evaluated in concrete specimens were pulse velocity and residual elasticity modulus by ultrasound, compressive strength, fracture energy and residual flexural strength. These properties were assessed in prismatic specimens, cast for three-point bending tests (TPB). Given that the concrete with 10 kg/m^3^ (HF10) was considered as structural material (based on the use of the same mix proportioning and fibres used in references [9,17]), several additional temperatures were chosen between 150 °C and 200 °C for applying to the specimens made with this material. This was made in order to find the temperature at which the structural capacities were strongly affected. This can be of relevance for the structural design of PFRC elements. Thus, HF10 specimens were exposed to 150 °C, 165 °C, 175 °C, 185 °C and 200 °C.

The fracture tests were performed following RILEM TC-187-SOC [18] with specimens of 100 × 100 × 430 mm^3^ (height × depth × length). In order to assess compressive strength tests, cubic specimens of side 10 mm were obtained by sawing them from the reaming halves of the prismatic specimens tested. For each temperature, a pair of cubic pieces were tested. The tests were performed according to EN 12390-3 [19]. Table 4 specifies the number of specimens tested in fracture for each concrete type.

The heating process of specimens was carried out by means of convection heat in an oven, at an approximate heating rate of 2.80 °C/min. When the specimens reached the maximum temperature of analysis, they remained for 3 h at such temperature. The oven turned on and off using programmers during the night before the test day. The test specimens cooled on the stove for a period of 7 h.

The heating time of the concrete specimens was chosen based on references [20,21] that used heated rates in the interval 1 °C/min to 10 °C/min, during 1 to 3 h at maximum temperature. In addition, the campaign carried out by Josef Novak [22] was considered. In this reference, the temperature was monitored externally and in the core of cubic specimens of 15 × 15 × 15 cm^3^ by using thermocouples, showing that the heating times chosen were adequate.

#### 2.2.1. Residual Pulse Velocity

Ultrasound pulse velocity test may allow the evaluation of porosity and internal damage of concrete. The tests were performed before and after exposing the specimens to the heating process. In the case of the measurements after heating, they were performed when the specimen reached 30 °C in order to avoid wrong lectures during the test [23].

For all the specimens, pulse velocity was assessed according to the methodology proposed by standard UNE-EN 12504-4: 2006 [24]. The pulse velocity for most of the specimens tested, ranged from 4000 m/s to 4600 m/s, being good to excellent concrete according to the Leslie and Cheesman classification [23]. This test was performed for all mixes, and the average values of pulse velocity as well as the residual modulus of elasticity, obtained through Equation (1), can be seen in Table 5 (HF), Table 6 (HF3) and Table 7 (HF10). The specimens were named by the sequence Concrete (HF, HF3, HF10) batch number of specimen. Pulse velocity (*Vp*) and residual modulus of elasticity (*E*) at 20 °C have been labelled in the tables as *Vp*_20_ and *E*_20_ respectively.

(1) $$E=v_{p}^{2}\cdot \rho$$

#### 2.2.2. Three-Point Bending Tests

**Figure 4:**
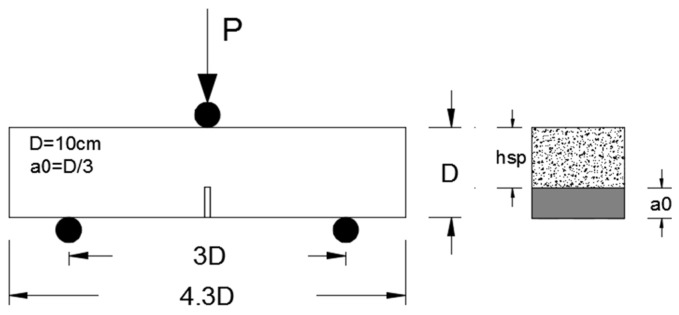
Scheme of the three-point bending test [17].

In order to obtain the residual flexural tensile strengths of PFRC, tests were carried out according to RILEM TC-187-SOC recommendation [18] and the residual strengths were computed according to UNE-EN 14561: 2007 standard [25]. This procedure has been accepted and was explained in detail in references [17,26]. The span and notch length corresponded to the specifications given by the RILEM TC-187-SOC [18], the span being equal to 3*D* (300 mm) and the length of the notch *D*/3 (33.3 mm), where *D* is the height of the specimen. Figure 4 specifies these dimensions, *a*_0_ being the length of the notch, *h_sp_* the ligament length and *P* the applied load. In addition to the residual strengths, the maximum load that corresponds to the limit of proportionality (*F_LOP_*), the deflection and crack opening were obtained by means of two Linear Variable Differential Transformer (LVDT) (to measure deflection in the midspan) and one Crack Mouth Opening Displacement (CMOD) resistive transducer (to measure the crack mouth opening), respectively. The two LVDTs were placed at each side of the specimen in the midspan and the deflection was considered the average value. The CMOD was placed in the lips of the notch in order to measure the opening of the notch.

With the results of the tests, the curves stress vs. crack opening (FL-CMOD) were obtained in order to determine the residual flexural tensile strengths for crack openings of 0.5, 1.5, 2.5 and 3.5 mm. The fracture energy was calculated with the load vs. deflection curves (Load–vertical displacement of the application load point).

It is worth noting that the careful manufacturing process, being of key importance in this study, in addition to the accuracy of the equipment and measuring devices permitted the reduction of the experimental scatter. The specimen positioning was carefully made by means of laser devices. The notch was machined with a water-cooled low-speed diamond cutting disc. The concrete specimens rested on two rigid steel cylinders laid on two ground supports, which allowed free rotation out of the plane of the beam and guarantee negligible friction rolling in the longitudinal direction of the beam. Thus, the results of the fracture tests showed a remarkably low degree of scatter that can be verified for the specimens exposed to 20 °C by previous works on references [6,9]. For the three most relevant temperatures (150 °C and 200 °C for HF3 and 150 °C, 165 °C and 200 °C for HF10) two specimens were tested. Moreover, expecting that HF10 would meet the structural requirements, some more specimens were produced. Such specimens were used in order to find the threshold at which the material behaved similar to the result at 200 °C. Thus, for 175 °C and 185 °C one specimen was tested.

#### 2.2.3. Fracture Energy

**Figure 5:**
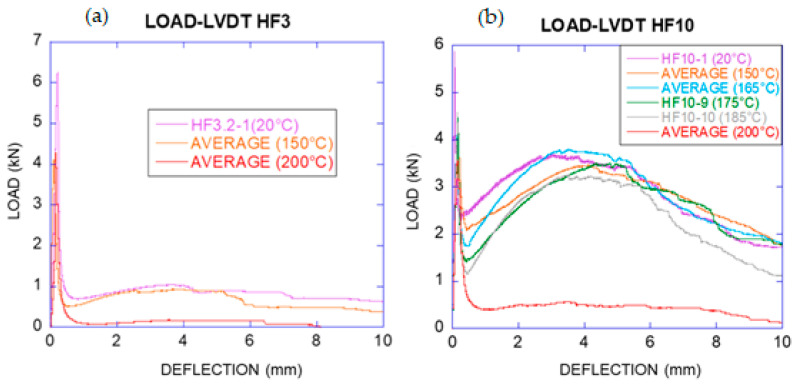
Load–deflection curves of (**a**) HF3 and (**b**) HF10 at various temperatures (average at each temperature).

Fracture energy was analysed for both plain and PFRC specimens. The load at the proportionality limit (*F_LOP_*) was reached in all cases for CMOD below 0.1 mm. The first post-cracking branch of the curve after *F_LOP_* followed the typical shape of the concrete softening behaviour up to CMOD values close to 0.5 mm (*F_MIN_*) at which a new reloading branch initiated. Such reloading branch was constantly increasing up to the crack opening (CMOD) at which the fibres collapse by any of the failure mechanisms (mainly sliding or breaking). Such point of the curve was named maximum residual remaining load (*F_REM_*). This can be better understood by seeing Figure 5. The values of fracture energy (*G_F_*) and coefficient of variation for plain concrete as a function of temperature are shown in Table 8, being the reference for the discussion of the results of PFRC.

The load–deflection curves of HF3 and HF10 exposed to temperatures ranging from 20 °C to 200 °C are shown in Figure 5. This shows that these tests revealed that the degradation process of PFRC is relatively stable, with very similar behaviour for temperatures up to 185 °C. Nevertheless, the curves of the specimens subjected to 200 °C showed a clear damage although there was a remaining load-bearing capacity that could be of interest.

It should be highlighted that the results of HF10 specimens showed that the contribution of the fibres was still significant at 165 °C. As can be seen in Figure 5, *F_REM_* of the average curve at 165 °C was even higher than that of 20 °C, with a residual percentage of load-bearing capacity of 103% respect to 20 °C. The fibres, as a part of fibre reinforced concrete, reduced the degradation of its properties by temperature if this is compared with the results of isolated fibres. At 175 °C, the description of the load–deflection curve continues with typical behaviour of the polyolefin fibres, with *F_REM_* remaining at 95% of the curve obtained for 20 °C. At 185 °C, *F_REM_* was 88% of the curve obtained for 20 °C, still being significant.

Conversely, load–deflection curve was different for 200 °C. Such a curve showed residual values for *F_LOP_*, *F_MIN_* and *F_REM_* of 61%, 17% and 16% respectively compared with the tests of the specimens at 20 °C. The load-bearing capacity decreased sharply and the loss of mechanical properties of the effective fibres in the section was evident. When *F_MIN_* was surpassed, it reduced the load value close to zero for a 10 mm deflection. The fracture energy (*G_F_*) assessed for HF3 and HF10 can be seen in Table 9 and Table 10 as well as the coefficient of variation.

#### 2.2.4. Residual Flexural Tensile Strengths

The structural capacity of PFRC was assessed according to UNE-EN 14561:2007 [25], EHE-08 and fib Model Code [27]. In such standards, it is set that in order to consider the contribution of the fibres in the structural design, the residual flexural tensile strengths *f_R_*_1_ (strength at CMOD 0.5 mm) should be superior to 40% of *f_LOP_* and *f_R_*_3_ (strength at CMOD 2.5 mm) shall not be less than 20% of *f_LOP_*. Thus, the analysis of residual flexural tensile strengths *f_R_*_1_, *f_R_*_2_, *f_R_*_3_, *f_R_*_4_ were considered with respect to the increase in temperature. Such residual strengths are those obtained through Equation (2) at CMOD values of 0.5 mm, 1.5 mm, 2.5 mm and 3.5 mm, respectively.

(2) $$f_{Ri}=\frac{3\cdot F_{i}\cdot span}{2\cdot depth\cdot hsp^{2}}$$

Given the low fibre dosage of HF3, the specimens did not meet the requirements of the standards even when exposed to ambient temperature. At least until temperatures of 150 °C the specimens behaved similarly to those only exposed to ambient temperature. In the case of the HF3 specimens exposed to 200 °C, the fibres that seemed to be closest to the surface reaching their melting point and the residual properties were strongly affected. The residual flexural tensile strengths of HF3 exposed to various temperatures are shown in Table 11.

The tests conducted on HF10 specimens showed that as the temperature of exposure increased, *f_LOP_* decreased with a value of 5.94 MPa in the control test to 4.22 MPa at 150 °C, a decrease of 29%. Regarding *f_R_*_1_ and *f_R_*_3_ the specimens met the structural requirements of the standards up to those exposed to 165 °C, with values 42% and 71% for *f_R_*_1_ and *f_R_*_3_ respectively. From 175 °C to 200 °C, only *f_R_*_3_ met the requirements. This is worth mentioning because such is the requirement for ultimate limit state in design. Table 12 presents the residual flexural tensile strengths of HF10 exposed to various temperature values.

#### 2.2.5. Compressive Strength

The variation of the compressive strength was assessed by testing at least three specimens, rejecting highly dispersed values and considering repeatability or reproducibility specified in EN 12390-3 [19]. The results obtained results for each concrete type can be seen in Table 13, Table 14 and Table 15.

## 3. Discussion

### 3.1. Uniaxial Tests of Isolated Fibers

#### 3.1.1. Elasticity Modulus and Maximum Tensile Strength

**Figure 6:**
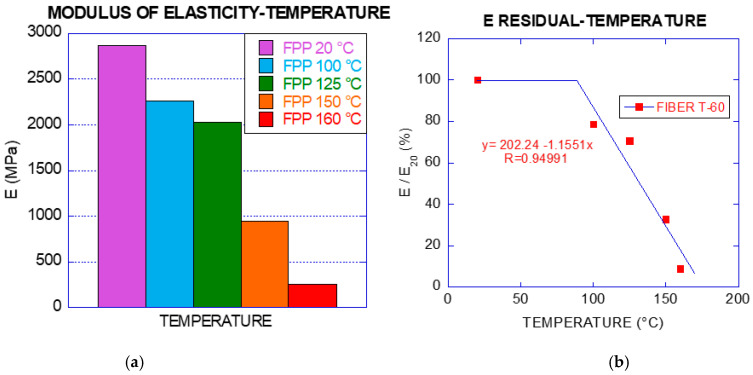
(**a**) Modulus of elasticity of polyolefin fibres in the uniaxial tensile tests; (**b**) residual modulus of elasticity in percentage of the modulus obtained for fibres only exposed to ambient temperature (the equation is the linear fitting of the inclined branch).

As can be seen in Figure 6, the reduction of the modulus of elasticity could be modelled through a simplified model as a bilinear behaviour between 20 °C and 160 °C. In such a simple model, the value of the modulus of elasticity could be considered constant up to 90 °C. The changes in the fibre were noticeable from 100 °C, especially at 110 °C as expected [15]. When the fibre exceeded 125 °C, its decrease was abrupt, adapting this scatter of points (from 100 to 160 °C) to a linear trend with negative slope.

**Figure 7:**
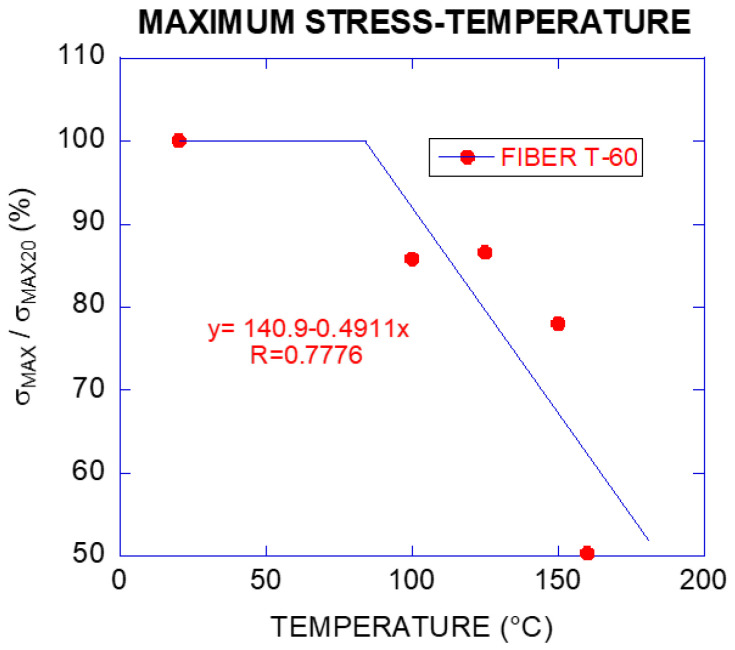
Maximum residual tensile strength (%) vs. temperature of fibre T-60 (the equation is the linear fitting of the inclined branch).

Specimens subjected to 150 °C and 160 °C showed residual values of the modulus of elasticity 33% and 9% lower respectively. Thus, after exposed to such temperatures the degradation was noticeable which could be attributed to the proximity to the crystalline melting temperature (175 °C) [28]. The symmetry of the homopolymer generates that molecules are able to be packaged in an ordered reticulated array [29] as the temperature increases so the fibre maintains its shape, but not its section and length. The maximum tensile strength vs. temperature was also adapted to a bilinear behaviour as it is presented in Figure 7.

The percentage of reduction of maximum tensile strength with increase of temperature was lower than in the case of the modulus of elasticity. The residual strengths for specimens subjected up to 150 °C maintained 78% of the value for the control specimens. The residual tensile strength for the specimens exposed to 160 °C was still 50% of the control specimens.

The polyolefin is a partially crystalline polymer, having in general greater mechanical strength than a polymer with an amorphous structure [30]. The fibre length reduction caused by temperature supposed an increment of the cross-sectional area. Maximum load values for each temperature tested ranged between 286.4 N and 239.3 N. Given the increase of the cross section, the results revealed that the maximum load value was obtained for fibres exposed to 150 °C, although the maximum stress was found for non-altered fibres, only exposed to 20 °C.

#### 3.1.2. Strain

**Figure 8:**
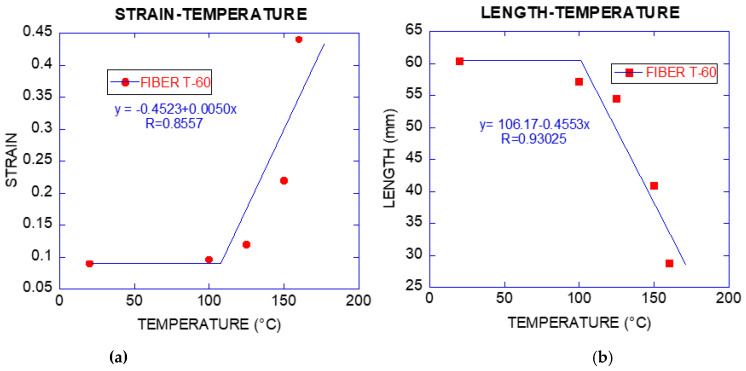
(**a**) Percentage of residual strain vs. Temperature fibre T-60; (**b**) length vs. Temperature fibre T-60 (the equations are the linear fittings of the inclined branches).

The deformation increased with temperature: at 100 °C and 125 °C the growth in deformation was about 1.06 and 1.32 times superior in reference to fibres only exposed to room temperature. These values increased sharply for fibres exposed to 150 °C and 160 °C, being 2.45 and 4.92, respectively. Figure 8 shows this behaviour. Higher capacity of deformation led to lower modulus of elasticity and stress–strain curves with lower slopes.

The elongation capacity of fibre is not affected by being exposed to increasing temperatures. According to the results obtained, the minimum and maximum elongation varies between 14.55 mm for 20 °C and 17.45 mm for 125 °C, oscillating between these values for the rest of temperatures.

This sudden increase of the strain could be attributed to the fact that the fibre diminishes its length although maintains its capacity of elongation in the tests. The fibre reduced its length down to 48% of its initial magnitude for 160 °C. Figure 8b shows this shortening of the fibres associated with temperature.

### 3.2. Pulse Velocity and Residual Elasticity Modulus

**Figure 9:**
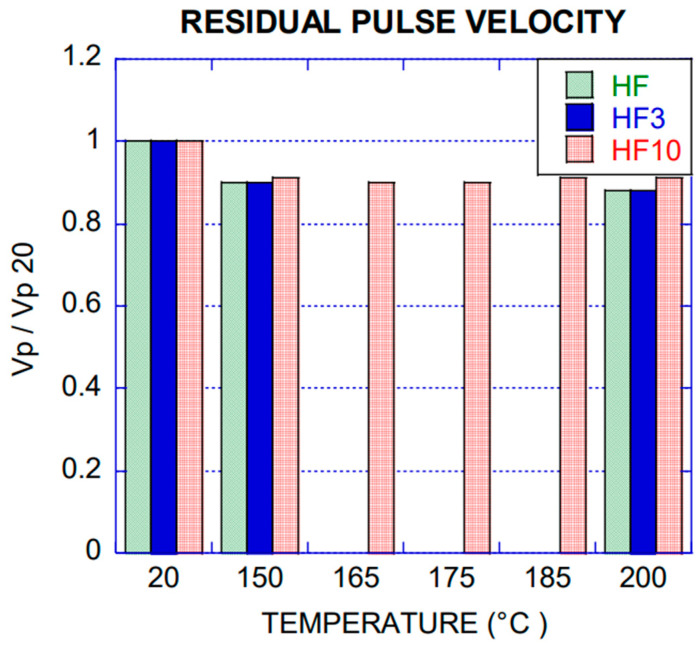
Residual pulse velocity collected by ultrasound test.

The residual pulse velocity for all the concrete types according to Figure 9 showed a reduction of 10% at 150 °C and almost constant up to 200 °C, attributed to damage by microcracking in the concrete matrix, product of decomposition of ettringite and CSH (Calcium silicate hydrate), characteristic phenomenon observed between 100–200 °C [31].

In all cases, values between 88% and 91% of pulse velocity at room temperature were obtained. These results were comparable with those evaluated by K.K. Sideris [20] for high strength concrete with addition of polypropylene fibres.

**Figure 10:**
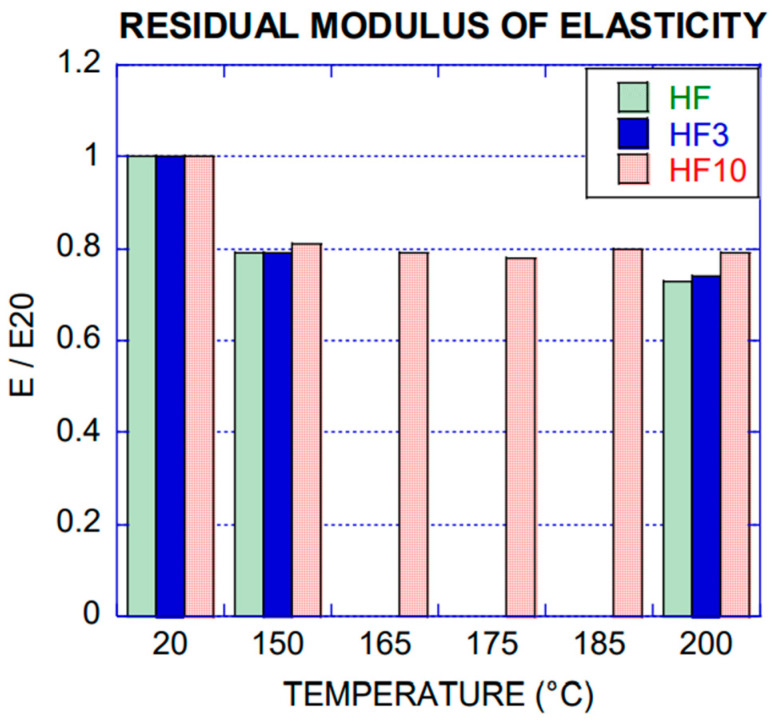
Residual modulus of elasticity collected by ultrasound test.

In reference to the static modulus of elasticity, it can be appreciated a similar behaviour to the previous results as shown in Figure 10. Due to the damage of the concrete microstructure, the modulus of elasticity decreased by 20%. Residual values ranged from 80% to 73% of the unexposed concrete for the specimens exposed to the range 150 to 200 °C.

### 3.3. Compressive Strength

**Figure 11:**
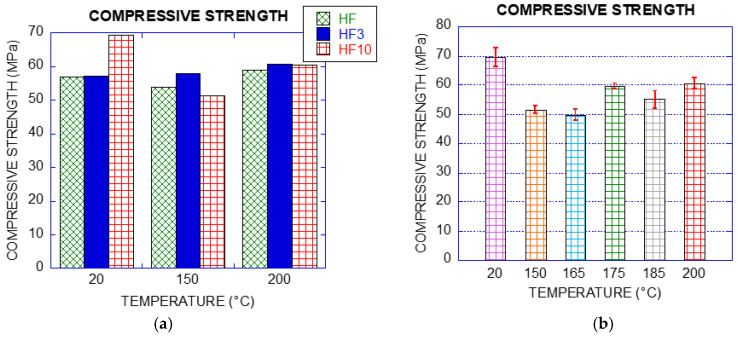
(**a**) Compressive strength of all mixes; (**b**) compressive strength HF10.

Figure 11 shows the compressive strength of all the concrete types. At room temperature, HF10 reached an average value of 69 MPa, being 20% higher than the other concrete types. For HF and HF10 strength values decreased at 150 °C being the residual magnitudes 95% and 74% respectively. Concrete with fibres at 200 °C showed a slight increase of the compression strength with respect to the residual values obtained at 150 °C, being 106% and 87% for HF3 and HF10, respectively. Such results are in accordance with Pliya [32], who observed a decrease in compressive strength at 150 °C and a gradual increase up to 300 °C (high strength concrete with steel fibres in proportion of 30 to 40 kg/m^3^).

In the case of HF10, specimens were exposed to several increasing steps of temperature between 150 °C and 200 °C, as can be seen in Figure 11b. The results show that although there was a noticeable decrease of strength at 150 °C, the compressive strength remained similar up to 165 °C and a slight increase took place for the specimens exposed to 175 °C, 185 °C and 200 °C.

### 3.4. Toughness Index and Fracture Energy

Toughness index was computed by dividing the area under load deflection curve for concrete HF3 and HF10 by the reference value of the energy absorption of the concrete without fibres (HF), obtained from the area under the Load–LVDT curve at 0.5 mm of deflection. The toughness index (*TI*) could be computed by expression (1), being *A_FRC_* the area under the load–deflection curve for HF3 and HF10 and *A_HF_* the area under the load–deflection curve for HF at 0.5 mm.

(3) $$TI=\frac{A_{FRC}}{A_{HF}}$$

**Figure 12:**
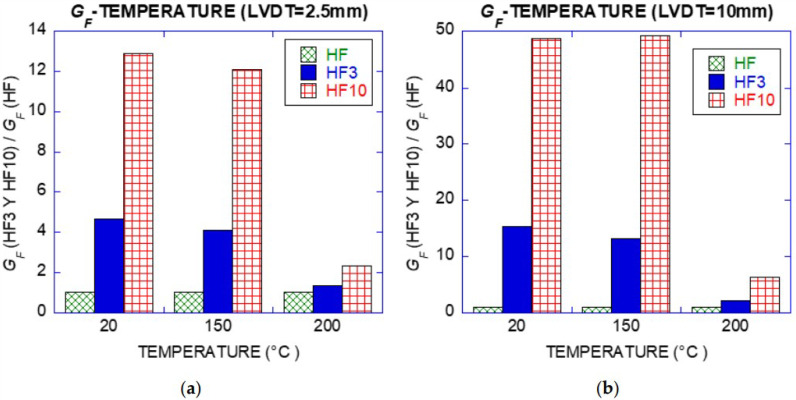
Fracture energy: (**a**) for 2.5 mm of midspan deflection; (**b**) for 10 mm of midspan deflection.

HF10 showed higher toughness index than HF3. As deflection increased, such values of HF10 reached 12 and 49 times higher at 2.5 mm and 10 mm as it is shown in Figure 12. For a deflection of 0.5 mm, the toughness index was 1.10 and 2.30 for HF3 and HF10 respectively, the concrete matrix being responsible for the flexural strength at this stage.

**Figure 13:**
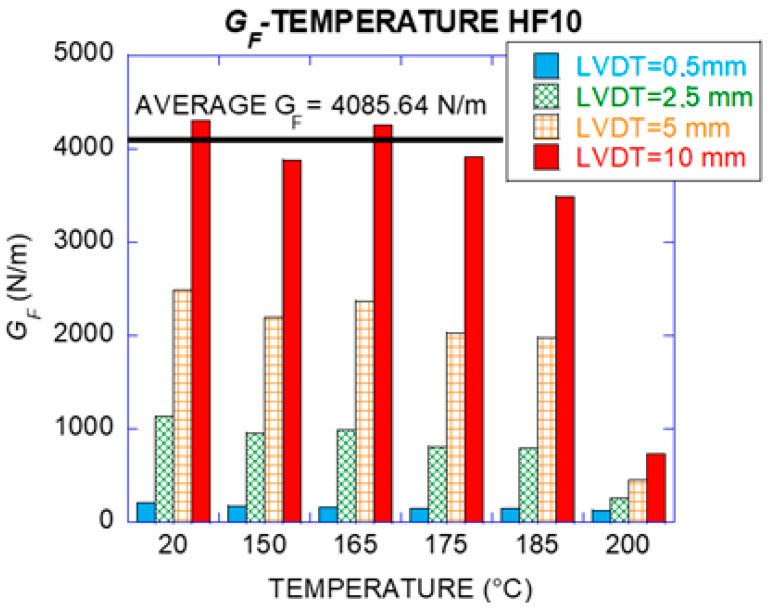
Fracture energy HF10 for various deflection values.

Figure 13 shows a detailed fracture energy analysis for HF10 exposed to various steps of temperature. Specimens exposed to 150 °C, 165 °C and 175 °C showed a very slight reduction of the fracture energy remaining around 90% of the reference (fracture energy at 20 °C). A slight increase at 165 °C was perceived, with a residual value of 99% that could be attributed to a greater number of effective fibres in the cracking section, 60 and 69 fibres. From 165 °C to 185 °C, the fracture energy showed a slight decrease though remaining a high degree of its load-bearing capacity. However, specimens exposed to 200 °C showed a remarkable descent of the fracture energy.

**Figure 14:**
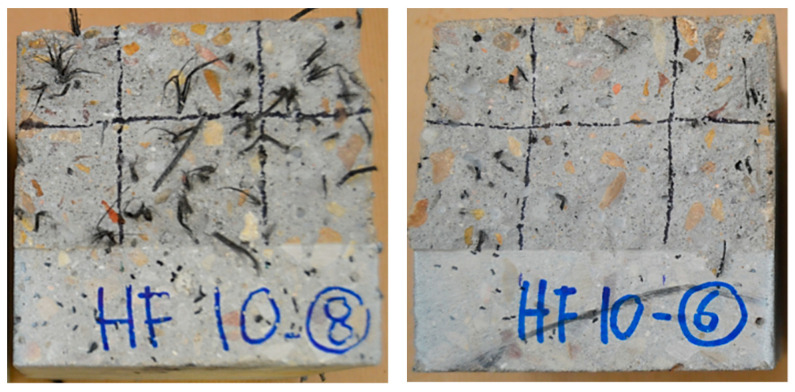
Number of fibres in the fracture surface of specimens exposed to 165 °C and 200 °C.

From 20 °C to 175 °C an average fracture energy value of 4086 N/m (94% of the fracture energy at room temperature) was assessed. At 185 °C, 81% of the fracture energy was kept and at 200 °C a residual value of 17% was found, attributed to the change in the state of the fibres. When they reach their melting point, the effective number of fibres in the fracture surface decreased, with a reduction of 50%. Figure 14 shows this reduction (from 62 fibres at 20 °C, to 31 fibres at 200 °C).

### 3.5. Fracture Surface Analysis

**Figure 15:**
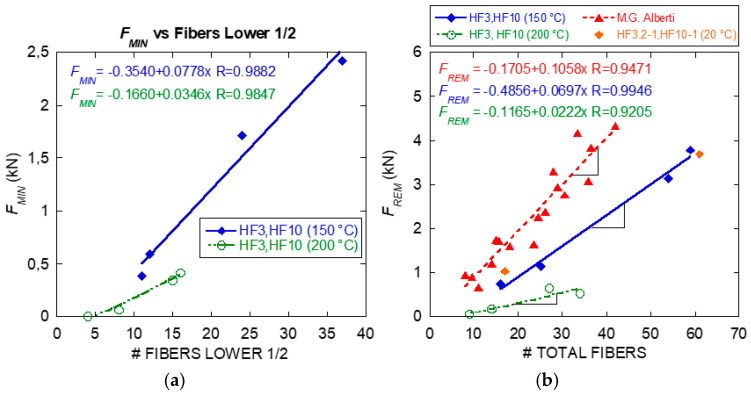
(**a**) *F_MIN_* vs. number of fibres lower 1/2, (**b**) *F_REM_* vs. total fibres.

To assess the fracture properties of the specimens manufactured with fibre reinforced concrete, a fracture surface analysis was performed following previously published research and models [33,34,35]. The total number of fibres in the fracture surface, as well as those placed in the lower half of the fracture surface (see delimitation lines in Figure 14), were counted for each specimen. The results are shown in Figure 15 with respect to the minimum post-cracking load (*F_MIN_*) and the maximum remaining post-cracking load (*F_REM_*). These values are the reference values to be used in the analysis of the fracture behaviour of PFRC [9,13]. Figure 15 shows that the trend of previous research was followed for specimens exposed to 20 °C. Likewise, the figure shows that remarkable correlations were found for specimens exposed to 150 °C and 200 °C although with lower slopes in the linear fittings.

### 3.6. Residual Flexural Tensile Strengths

The fracture behaviour of fibre reinforced concrete was assessed. This analysis is of key relevance in order to assess the structural contribution of the fibres to Service Limit State and Ultimate Limit State (SLS and ULS, respectively). According to annex 14 of EHE-08 [36], residual strengths at crack openings of 0.5 mm (*f_R_*_1_) and 2.5 mm (*f_R_*_3_) are the parameters associated with structural design. Therefore, the evolution of such parameters when PFRC is exposed to high temperature is directly related with the residual strengths that could be considered for structural design at each temperature step.

**Figure 16:**
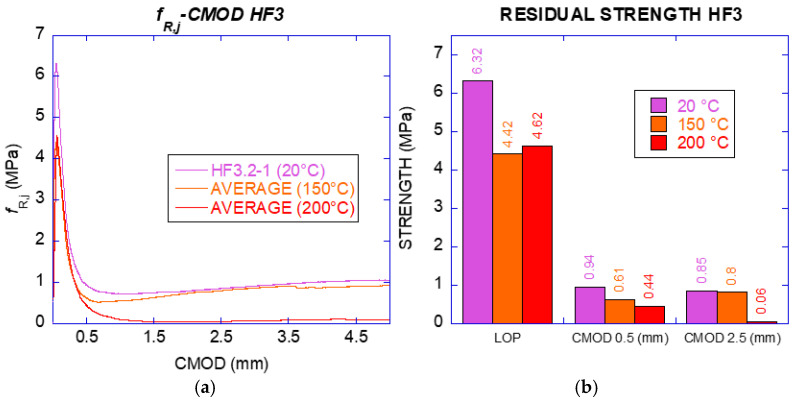
(**a**) Curve Strength -CMOD (HF3); (**b**) residual strength for CMOD required by the standard (HF3).

The residual strengths of HF3 were below the required by the standards at any of the temperatures tested. The fracture curves can be seen in Figure 16. The values of *f_R_*_1_ and *f_R_*_3_ of the specimens exposed only to room temperature were about 15% of the strength at limit of proportionality (*f_LOP_*) and are detailed in Table 11. The performance was similar for those specimens exposed to 150 °C and 200 °C. Such residual values were 9% (*f_R_*_1_) and 1% (*f_R_*_3_).

**Figure 17:**
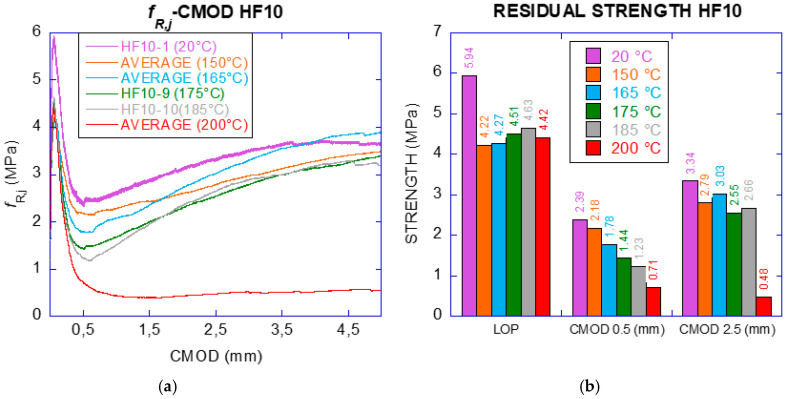
(**a**) Curve Strength-CMOD (HF10); (**b**) residual strength for CMOD required by the standard (HF10)

The behaviour of HF10 specimens is shown in Figure 17. The most relevant residual strengths for the specimens exposed to each temperature can be seen in Table 12. Considering the main residual strengths in the standards, specimens without exposure to high temperature showed percentages of 40% at 0.5 mm of CMOD (*f_R_*_1_) and 56% at 2.5 mm of CMOD (*f_R_*_3_) with respect to a value of 5.94 MPa of *f_LOP_*. This behaviour, considered by the standards as structural, is even more noticeable for the specimens exposed to 150 °C and 165 °C. Such specimens reached 52% and 42% in *f_R_*_1_, and for *f_R_*_3_ the percentages reached 66% and 71% respectively.

For higher steps of temperature, the SLS requirement is not met (*f_R_*_1_ was lower than 40%), being 32% of *f_LOP_* for 175 °C, although the material maintained the main characteristics of structural fibre reinforced concrete with 57% of *f_LOP_* in the case of *f_R_*_3_ (value of reference for ULS). As shown in the results, the performance for ULS was still acceptable at 175 °C and 185 °C with residual strengths of 2.55 MPa and 2.66 MPa, respectively. Nevertheless, a strong reduction was found for specimens subjected to 200 °C, with absence of any structural behaviour although still showing structural integrity as compared with plain concrete.

## 4. Conclusions

The use of polyolefin fibre reinforced concrete has become an alternative to steel fibres. However, the main possible drawback of this type of fibres could be their behaviour when exposed to high temperature given their low melting temperature. The main aim of this research was to assess the behaviour of PFRC specimens as well as PF when they are exposed to temperatures ranging from 100 °C to 200 °C. Among the results, the variations of the residual strengths of structural PFRC specimens subjected to elevated temperatures may be useful for designing structures with PFRC that can be exposed to such conditions.

The use of polymer structural fibres can be more adequate than other structural ones for reinforcing the concrete lining of tunnels, protection walls in industrial plants, structures subjected to marine environments or port pavements. The use in such applications requires assessing their structural behaviour when subjected to high temperatures.

This research shows that up to 150 °C the structural behaviour and the fracture energy of PFRC specimens were not affected. Between 150 °C and 200 °C, some properties were deteriorated and at 200 °C a significant percentage of the fibres were molten.

In the fibre reinforced concrete specimens exposed to 150 °C, the damage occurred mainly in the concrete matrix, causing a decrease in the compressive strength, static modulus of elasticity and ultrasonic pulse velocity. The modulus of elasticity and ultrasonic pulse velocity maintained similar values up to 200 °C, while compressive strengths remained in similar values.

PFRC specimens with 10 kg/m^3^ of PF showed that even after exposure up to 165 °C they met the required structural contributions considered by the standards (a remaining residual strength of 103% stood out with respect to the result for specimens only exposed to room temperature). Conversely, specimens exposed to 200 °C showed absence of any structural capacity, though the specimens still did not collapse or show brittle failure.

Based on this study, it is suggested to perform an evaluation of spalling behaviour, and mechanical properties of reinforced concrete with polyolefin fibres at temperatures above 200 °C, so the possibilities of PF could be explored for temperatures beyond such a temperature.

Both mechanical and physical properties (modulus of elasticity, maximum tension, deformation, length) of the fibres studied individually exposed to high temperatures showed a bilinear behaviour, with a negative slope for all of them except in the case of deformation, which was positive. The influence of temperature was visible from 100 °C and showed and abrupt increase in the deterioration of the properties from 150 °C.

## Figures and Tables

**Table 1:** Mechanical properties of fibres used in experimental campaign [15].

SIKA FIBER T-60	
Density	0.92 g/cm^3^
Length of fibre	60 mm
Tensile strength	560 MPa
Distortion temperature	110 °C
Melting point	280 °C
Modulus of elasticity	>9 GPa

**Table 2:** Physical and mechanical properties polyolefin fibres after being exposed to high temperatures (average value of five specimens).

Polyolefin Fibres and Temperature	Length	Width	Thickness	Max. Load	Max. Stress	Max. Strain	Modulus of Elasticity
	(mm)	(mm)	(mm)	N	(MPa)	(%)	(MPa)
FPP-20 °C	60.31	1.51	0.67	264	259.44	9	2874
FPP-100 °C	57.12	1.61	0.69	243	222.79	10	2262
FPP-125 °C	54.47	1.64	0.70	256	224.63	12	2029
FPP-150 °C	40.86	1.82	0.78	286	202.28	22	943
FPP-160 °C	28.69	2.12	0.87	239	130.82	44	257

**Table 3:** Mix proportions used concrete in experimental campaign [17].

Concrete Formulation	Cement (kg/m^3^)	Limestone Powder (kg/m^3^)	Water (kg/m^3^)	Sand (kg/m^3^)	Gravel (kg/m^3^)	Grit (kg/m^3^)	Superplasticiser (% Cement Weight)	Polyolefin Fibres (kg/m^3^)
HF	375	100	187.5	916	300	450	0.75	-
HF3	375	100	187.5	916	300	450	0.75	3
HF10	375	100	187.5	916	300	450	0.82	10

**Table 4:** Tested specimens in concrete experimental campaign.

Nomenclature	Fibre Admixture (kg/m^3^)	Fibre Length (mm)	Length (mm)	Width (mm)	Height (mm)	No. of Specimens
**HF**	-	60	430	100	100	6
**HF3**	3	60	430	100	100	5
**HF10**	10	60	430	100	100	9

**Table 5:** Pulse velocity (*Vp*) and residual modulus of elasticity (*E*) of HF specimens before and after heating.

Temp.	Specimen	Before Heating			After Heating			*E*/*E*_20_	*Vp*/*Vp*_20_
		*Vp* (m/s)	*ρ* (kg/m^3^)	*E* (MPa)	*Vp* (m/s)	*ρ* (kg/m^3^)	*E* (MPa)		
20 °C	HF 1-1	4728.5	2287.0	51,135.2	-	-	-	100.0%	100.0%
		(0.004) *							
	HF 2-1	4665	2298.6	50,022.9	-	-	-	100.0%	100.0%
		(0.000) *							
150 °C	HF 1-2	4708	2276.6	50,461.9	4250.5	2225.7	40,211.1	79.7%	90.0%
		(0.002) *			(0.004) *				
	HF 2-2	4670	2314.6	50,478.5	4193	2258.6	39,708.4	78.7%	90.0%
		(0.002) *			(0.001) *				
200 °C	HF 1-3	4710.5	2317.8	51,429.8	4103	2213.9	37,270.0	72.5%	87.0%
		(0.002) *			(0.002) *				
	HF 2-3	4634	2270.8	48763.8	4079.5	2164.8	36,027.5	73.9%	88.0%
		(0.002) *			(0.001) *				

* Coefficients of variation of between two measures of pulse velocity.

**Table 6:** Pulse velocity (*Vp*) and residual modulus of elasticity (*E*) of HF3 specimens before and after heating.

Temp.	Specimen	Before Heating			After Heating			*E*/*E*_20_	*Vp*/*Vp*_20_
		*Vp* (m/s)	*ρ* (kg/m^3^)	*E* (MPa)	*Vp* (m/s)	*ρ* (kg/m^3^)	*E* (MPa)		
20 °C	HF 3.1-1	4644	2255.1	48,635.0	-	-	-	100.0%	100.0%
		(0.002) *							
	HF 3.2-1	4683	2310.2	50,663.5	-	-	-	100.0%	100.0%
		(0.005) *							
150 °C	HF 3.1-2	4685	2272.7	49,883.7	4236	2213.0	39,708.7	79.6%	90.0%
		(0.002) *			(0.002) *				
	HF 3.2-2	4667.5	2261.3	49,264.6	4182	2205.3	38,569.2	78.3%	90.0%
		(0.002) *			(0.001) *				
200 °C	HF 3.1-3	4675	2273.6	49,691.2	4126.5	2164.6	36,858.5	74.2%	88.0%
		(0.000) *			(0.003) *				
	HF 3.2-3	4655	2271.5	49,221.8	4075.5	2162.5	35,918.5	73.0%	88.0%
		(0.002) *			(0.001) *				

* Coefficients of variation of between two measures of pulse velocity.

**Table 7:** Pulse velocity (*Vp*) and residual modulus of elasticity (*E*) of HF10 specimens before and after heating.

Temp.	Specimen	Before Heating			After Heating			*E*/*E*_20_	*Vp*/*Vp*_20_
		*Vp* (m/s)	*ρ* (kg/m^3^)	*E* (MPa)	*Vp* (m/s)	*ρ* (kg/m^3^)	*E* (MPa)		
20 °C	HF 10-1	4610	2296.7	48,809.0	-	-	-	100.0%	100.0%
		(0.002) *							
150 °C	HF 10-3	4459.5	2252.8	44,801.3	4031.5	2213.2	35,971.0	80.3%	90.0%
		(0.004) *			(0.003) *				
150 °C	HF 10-5	4426.5	2264.6	44,372.0	4013	2228.2	35,884.0	80.9%	91.0%
		(0.004) *			(0.001) *				
165 °C	HF 10-4	4582.5	2278.5	47,846.3	4113	2227.5	37,682.9	78.8%	90.0%
		(0.002) *			(0.001) *				
165 °C	HF 10-8	4397	2219.0	42,900.9	3945.5	2167.8	33,746.5	78.7%	90.0%
		(0.002) *			(0.001) *				
175 °C	HF 10-9	4571.5	2269.0	47,418.6	4095	2211.6	37,085.9	78.2%	90.0%
		(0.001) *			(0.001) *				
185 °C	HF 10-10	4451	2205.1	43,686.0	4041	2135.6	34,874.5	79.8%	91.0%
		(0.002) *			(0.000) *				
200 °C	HF 10-6	4442	2235.0	44,098.7	4043.5	2150.0	35,152.3	79.7%	91.0%
		(0.005) *			(0.003) *				
200 °C	HF 10-7	4470.5	2252.6	45,019.7	4025.5	2169.6	35,157.0	78.1%	90.0%
		(0.002) *			(0.002) *				

* Coefficients of variation of between two measures of pulse velocity.

**Table 8:** Fracture energy (*G_F_*) and coefficient of variation of HF specimens.

FRACTURE ENERGY AND COEFFICIENT OF VARIATION (N/m)					
Description		Specimen Dimension			LVDT = 0.4 mm
Temperature	Concrete	*B* (mm)	*D* (mm)	*a_o_* (mm)	*G_F_* (N/m)
20 °C	HF	100	100	33.33	88.44
					**(0.09)**
150 °C	HF	100	100	33.33	78.86
					**(0.12)**
200 °C	HF	100	100	33.33	115.12
					**(0.16)**

**Table 9:** Fracture energy *(G_F_)* of HF3 specimens.

FRACTURE ENERGY AND COEFFICIENT OF VARIATION (N/m)					
Description	Deflection (LVDT Measurement)				
	0.5 mm	1 mm	2.5 mm	5 mm	FINAL
Temperature	*G_F_* (N/m)	*G_F_* (N/m)	*G_F_* (N/m)	*G_F_* (N/m)	*G_F_* (N/m)
20 °C	175	229	415	776	1352
	-	-	-	-	-
150 °C	112	152	323	659	1035
	(0.19)	(0.23)	(0.27)	(0.30)	(0.47)
200 °C	131	141	153	188	230
	(0.13)	(0.10)	(0.02)	(0.16)	(0.19)

**Table 10:** Fracture energy *(G_F_)* of HF10 specimens.

FRACTURE ENERGY AND COEFFICIENT OF VARIATION (N/m)					
Description	Deflection (LVDT Measurement)				
	0.5 mm	1 mm	2.5 mm	5 mm	FINAL
Temperature	*G_F_* (N/m)	*G_F_* (N/m)	*G_F_* (N/m)	*G_F_* (N/m)	*G_F_* (N/m)
20 °C	207	407	1140	2479	4308
	-	-	-	-	-
150 °C	183	351	954	2196	3876
	(0.12)	(0.17)	(0.16)	(0.15)	(0.19)
165 °C	169	325	993	2377	4251
	(0.12)	(0.09)	(0.08)	(0.00)	(0.05)
175 °C	152	274	810	2037	3908
	-	-	-	-	-
185 °C	142	254	803	1981	3488
	-	-	-	-	-
200 °C	131	165	268	457	727
	(0.14)	(0.17)	(0.12)	(0.12)	(0.10)

**Table 11:** Residual flexural tensile strengths of HF3 exposed to various temperatures.

Residual Flexural Tensile Strengths (MPa) and Coefficient of Variation										
Description		*f_LOP_*	CMOD = 0.5 mm		CMOD = 1.5 mm		CMOD = 2.5 mm		CMOD = 3.5 mm	
Temperature	Concrete		*f_R_*_1_	%	*f_R_*_2_	%	*f_R_*_3_	%	*f_R_*_4_	%
20 °C	HF3	6.32	0.94	15%	0.74	12%	0.85	14%	0.97	15%
		----	----		----		----		----	
150 °C	HF3	4.42	0.61	14%	0.62	14%	0.80	18%	0.91	21%
		(0.01)	(0.23)		(0.36)		(0.31)		(0.31)	
200 °C	HF3	4.62	0.43	9%	0.04	1%	0.05	1%	0.08	2%
		(0.09)	(0.37)		(1.10)		(1.20)		(0.86)	

**Table 12:** Residual flexural tensile strengths HF10 exposed to various temperatures.

Residual Flexural Tensile Strengths (MPa) and Coefficient of Variation										
Description		*f_LOP_*	CMOD = 0.5 mm		CMOD = 1.5 mm		CMOD = 2.5 mm		CMOD = 3.5 mm	
Temperature	Concrete		*f_R_*_1_	%	*f_R_*_2_	%	*f_R_*_3_	%	*f_R_*_4_	%
20 °C	HF10	5.94	2.39	40%	2.87	48%	3.34	56%	3.64	61%
		----	----		----		----		----	
150 °C	HF10	4.22	2.18	52%	2.43	58%	2.79	66%	3.13	74%
		(0.05)	(0.23)		(0.19)		(0.15)		(0.13)	
165 °C	HF10	4.27	1.78	42%	2.35	55%	3.03	71%	3.55	83%
		(0.04)	(0.05)		(0.02)		(0.02)		(0.04)	
175 °C	HF10	4.51	1.44	32%	2.00	44%	2.55	57%	2.99	66%
		----	----		----		----		----	
185 °C	HF10	4.63	1.23	27%	1.96	42%	2.66	57%	3.01	65%
		----	----		----		----		----	
200 °C	HF10	4.42	0.71	16%	0.40	9%	0.48	11%	0.51	12%
		(0.08)	(0.23)		(0.14)		(0.02)		(0.09)	

**Table 13:** Compressive strength of HF specimens.

		*fcm* (MPa)	COV	*fcm* Residual (%)
Temperature 20 °C	HF	56.9	(0.06)	100%
Temperature 150 °C	HF	53.9	(0.04)	95%
Temperature 200 °C	HF	58.9	(0.07)	104%

**Table 14:** Compressive strength of HF3 specimens.

DESCRIPTION		*fcm* (MPa)	COV	*fcm* Residual (%)
Temperature 20 °C	HF 3	57.4	(0.05)	100%
Temperature 150 °C	HF 3	58.1	(0.03)	101%
Temperature 200 °C	HF 3	60.7	(0.02)	106%

**Table 15:** Compressive strength of HF10 specimens.

DESCRIPTION		*fcm* (MPa)	COV	*fcm* Residual (%)
Temperature 20 °C	HF 10	69.4	(0.05)	100%
Temperature 150 °C	HF 10	51.4	(0.03)	74%
Temperature 165 °C	HF 10	49.7	(0.04)	72%
Temperature 175 °C	HF 10	59.5	(0.02)	86%
Temperature 185 °C	HF 10	55.0	(0.05)	79%
Temperature 200 °C	HF 10	60.5	(0.03)	87%

## Data Availability

The data presented in this study are available on request from the corresponding author.

## References

[1] Bazant Z.P., Kaplan M.F. (1996). Concrete at High Temperatures: Material Properties and Mathematical Models.

[2] Varona F.B., Baeza F.J., Bru D., Ivorra S. (2018). Evolution of the bond strength between reinforcing steel and fibre reinforced concrete after high temperature exposure. Constr. Build. Mater..

[3] Liu X., Ye G., De Schutter G., Yuan Y., Taerwe L. (2008). On the mechanism of polypropylene fibres in preventing fire spalling in self-compacting and high-performance cement paste. Cem. Concr. Res..

[4] Sanjayan G., Stocks L.J. (1993). Spalling of high-strength silica fume concrete in fire. ACI Mater. J..

[5] Chen B., Liu J. (2004). Residual strength of hybrid-fiber-reinforced high-strength concrete after exposure to high temperatures. Cem. Concr. Res..

[6] Yermak N., Pliya P., Beaucour A.L., Simon A., Noumowé A. (2017). Influence of steel and/or polypropylene fibres on the behaviour of concrete at high temperature: Spalling, transfer and mechanical properties. Constr. Build. Mater..

[7] Varona F., Baeza F., Bru D., Ivorra S. (2018). Influence of high temperature on the mechanical properties of hibryd fibre reinforced normal and high strength concrete. Constr. Build. Mater..

[8] Park J.J., Yoo D.Y., Kim S., Kim S.W. (2019). Benefits of synthetic fibers on the residual mechanical performance of UHPFRC after exposure to ISO standard fire. Cem. Concr. Compos..

[9] Alberti M.G., Enfedaque A., Gálvez J.C. (2014). On the mechanical properties and fracture behavior of polyolefin fiber-reinforced self-compacting concrete. Constr. Build. Mater..

[10] Picazo A., Gálvez J.C., Alberti M.G., Enfedaque A. (2018). Assessment of the shear behaviour of polyolefin fibre reinforced concrete and verification by means of digital image correlation. Constr. Build. Mater..

[11] Blanco A., Pujadas P., De la Fuente A., Cavalaro S., Aguado A. (2013). Application of constitutive models in European codes to RC–FRC. Constr. Build. Mater..

[12] Alberti M.G., Enfedaque A., Gálvez J.C., Pinillos L. (2017). Structural Cast-in-Place Application of Polyolefin Fiber–Reinforced Concrete in a Water Pipeline Supporting Elements. J. Pipeline Syst. Eng..

[13] Alberti M.G., Enfedaque A., Gálvez J.C. (2015). Improving the Reinforcement of Polyolefin Fiber Reinforced Concrete for Infrastructure Applications. Fibers.

[14] Behfarnia K., Behravan A. (2014). Application of high performance polypropylene fibers in concrete lining of water tunnels. Mater. Des..

[15] Sika (2017). Sika-fiber T-60. Macrofibras Sintéticas con Carácter Estructural para el Refuerzo de Hormigones.

[16] UNE-EN 6892-1:2017 (2017). Metallic Materials—Tensile Testing—Part 1: Method of Test at Room Temperature (ISO 6892-1:2016).

[17] Alberti M.G. (2015). Polyolefin Fibre-Reinforced Concrete: From Material Behaviour to Numerical and Design Considerations. Doctoral Thesis.

[18] Planas J., Guinea G., Gálvez J., Sanz B., Fathy A. (2007). Indirect Test for Stress-Crack Opening Curve, de Experimental Determination of the Stress-Crack Opening Curve for Concrete in Tension—Final Report of RILEM Technical Committee TC 187-SOC.

[19] UNE-EN 12390-3 (2003). Testing Hardened Concrete—Part 3: Compressive Strength of Test Specimens.

[20] Sideris K.K. (2009). Performance of thermally damaged fibre reinforced concretes. Constr. Build. Mater..

[21] Chen B., Wu K., Yao W. (2004). Conductivity of carbon fiber reinforced cement-based composites. Cem. Concr. Compos..

[22] Novak J. (2017). Fire response of Hybrid Fiber Reinforced Concrete to High Temperature. Procedia Eng..

[23] Llopis V.P. (2014). Ensayos no Destructivos en Hormigón. Georadar y Ultrasonidos.

[24] UNE-EN 12504-4 (2006). Testing Concrete—Part 4: Determination of Ultrasonic Pulse Velocity.

[25] UNE-EN-14651:2007+A1 (2008). Test Method for Metallic Fibre Concrete—Measuring the Flexural Tensile Strength (Limit of Proportionality (LOP), Residual).

[26] Kawashima K., Zafra R., Sasaki K.K.T., Nakayama M. (2011). Effect of Polypropylene Fiber Reinforced Cement Composite and Steel Fiber Reinforced Concrete for Enhancing the Seismic Performance of Bridge Columns. J. Earthq. Eng..

[27] (2010). Fib Model Code 2010 (MC2010).

[28] Textos científicos.com. https://www.textoscientificos.com/polimeros/temperatura.

[29] Banthia N., Gupta R. (2006). Influence of polypropylene fiber geometry on plastic shrinkage cracking in concrete. Cem. Concr. Res..

[30] Fanella C., Naaman A. (1985). Stress-strain properties of fiber reinforced mortar in compression. ACI J..

[31] Pimienta P. (2017). Behaviour of high-performance concrete at high temperatures: Some highligths. RILEM Tech. Lett..

[32] Pliya P., Beaucour A.L., Noumowé A. (2011). Contribution of cocktail of polypropylene and steel fibres in improving the behaviour of high strength concrete subjected to high temperature. Constr. Build. Mater..

[33] Dupont D., Vandewalle L. (2005). Distribution of steel fibres in rectangular sections. Cem. Concr. Compos..

[34] Alberti M.G., Enfedaque A., Gálvez J.C. (2016). On the prediction of the orientation factor and fibre distribution of steel and macro-synthetic fibres for fibre-reinforced concrete. Cem. Concr. Compos..

[35] Alberti M.G., Enfedaque A., Gálvez J.C. (2018). A review on the assessment and prediction of the orientation and distribution of fibres for concrete. Compos. Part B Eng..

[36] EHE-08 (2008). Spanish Structural Concrete Code.

